# Evans Syndrome in Childhood: Long Term Follow-Up and the Evolution in Primary Immunodeficiency or Rheumatological Disease

**DOI:** 10.3389/fped.2019.00304

**Published:** 2019-07-23

**Authors:** Beatrice Rivalta, Daniele Zama, Giovanni Pancaldi, Elena Facchini, Maria Elena Cantarini, Angela Miniaci, Arcangelo Prete, Andrea Pession

**Affiliations:** Department of Pediatrics, Sant'Orsola-Malpighi Hospital, University of Bologna, Bologna, Italy

**Keywords:** Evans syndrome, immune cytopenias, children, primary immunodeficiency, autoimmune disease, rheumatological disease, refractory cytopenia, immunological characterization

## Abstract

Evans syndrome (ES) is a rare but challenging condition, characterized by recurrent and refractory cytopenia episodes. Recent discoveries highlighted that an appropriate diagnostic workup is fundamental to identify an underlying immune dysregulation such as primary immunodeficiencies or a rheumatological disease. We hereby describe clinical features and laboratory results of 12 pediatric patients affected by ES referred to the Pediatric Onco-Hematology Unit of Bologna. Patients experienced a median of four acute episodes of cytopenia with 9 years as median age at the onset of symptoms. In 8/12 (67%) patients an underlying etiology, primary immunodeficiencies, or rheumatological disease was identified. In 4/12 children, other immune manifestations were associated (Thyroiditis, Celiac disease, Psoriasis, Vitiligo, Myositis, Membranoproliferative Glomerulonephritis). ES remained the primary diagnosis in four patients (33%). At a median follow-up time of 4 years, 5/12 (42%) patients revealed a chronic ITP, partially responsive to second line therapy. Immunoglobulin Replacement Therapy (IRT) was effective with a good hematological values control in three patients with a secondary ES (ALPS, CVID, and a patient with Rubinstein Taybi Syndrome and a progressive severe B cell deficiency with hypogammaglobulinemia). Our experience highlights that, in pediatric patients, ES is often only the first manifestation of an immunological or rheumatological disease, especially when cytopenias are persistent or resistant to therapy, with an early-onset or when are associated with lymphadenopathy.

## Introduction

Evans Syndrome (ES) is a rare condition, first described by Evans in 1951 (1) as the association of Autoimmune Hemolytic Anemia (AIHA) and Immune Thrombocytopenia (ITP) and it was considered an idiopathic autoimmune disease. Until now different definitions for ES have been used in literature. Some authors defined ES as the presence of an autoimmune destruction of at least 2 cell lineages, while others requested the presence of AIHA and ITP regardless of autoimmune neutropenia (AIN). Others restricted diagnosis of ES only to patients with no identifiable underlying cause of AIHA with ITP (2, 3). Finally, there were authors considering secondary forms too (4, 5), most commonly primary immunodeficiencies (PID) and rheumatological disease. Fischer et al. reported a risk of autoimmune cytopenia 120 times higher in patients with PID than general population (6). Most frequently, in patients affected by PID, causes of ES were Common Variable Immunodeficiency (CVID) and Autoimmune Lymphoproliferative Syndrome (ALPS) (7, 8) whereas in rheumatological disease, Systemic Lupus Erythematosus (SLE) and Antiphospholipid Antibody Syndrome (APS) (9). For that reason, some authors considered useful screening for ALPS, CVID, SLE, and APS in children with chronic single lineage autoimmune cytopenia or multi-lineage autoimmune cytopenias and search for HIV infection in adolescents (4, 10). Hematological manifestations often precede the whole clinical onset of the immunodeficiency and it is only with further investigation that primary immunodeficiency is revealed. We hypothesized that recognition of an immunological background and of the cause of secondary ES could be useful to assess prognosis correctly and necessary for a proper therapeutic choice, especially regarding immunomodulators and corticosteroid-sparing agents (4, 8, 11). Although literature suggests an association of ES with primary immune dysregulation, data mainly come from small cohorts of adult patients. Considering that characteristics and outcomes of children with ES are not well-documented, this study aims to describe natural history of autoimmune multi-lineage cytopenias in children referred to our center.

## Materials and Methods

Between 2002 and 2018, 12 patients with Evans syndrome referred to the Pediatric Onco-Hematology Unit of Bologna and data regarding clinical history and immuno-autoimmune laboratory tests were retrospectively collected. Internal review board (IRB) approval was obtained at S. Orsola-Malpighi Hospital for the study. Written informed consent was previously obtained from children's parents. Population included pediatric patients (age 0–18 at onset of cytopenia) with at least two concurrent or sequential autoimmune cytopenias (ITP, AIHA, AIN). Secondary forms were included and idiopathic ES was considered as an exclusion diagnosis. According to international working group criteria, ITP diagnosis was based on a platelet count of <100 10^9^/L (12). AIHA was defined as anemia (Hb < −2SDS) and a positive direct antiglobulin test associated to signs of hemolysis (hemoglobin reductions, reticulocytosis, unconjugated hyperbilirubinemia, elevated lactate dehydrogenase, and low haptoglobin) (13). AIN was defined as an absolute neutrophil count <1.0 10^9^/L for 6 months after exclusion of other causes (e.g., drugs, infections, or known genetic mutation) (14). In secondary ES cases, diagnosis of immuno-rheumatological disorders was made according to recent literature. CVID was defined according to revised ESID diagnostic criteria^1^, ALPS according to 2009 NIH International workshop (15), Mixed Connective Tissue Disease (MCTD) according to recent literature (16), and SLE according to the SLE International Collaborating Clinics classification (17, 18). For each patient demographic data, clinical presentation, underlying diagnosis, treatments, and outcome were reported (Table 1). Lymphocyte subset, immunoglobulin dosages, screening for autoantibodies, and vaccine responses (Tetanus toxoid) were always collected. When ALPS was suspected, TCRα/β+CD4–CD8– Double Negative T cell (DNT) counts and vitamin B12 levels were investigated. *In vitro* assessment of Fas-mediated apoptosis was performed in patients with DNT > 2.5%, since it is considered a sensitive first–line screening test (19–21) (Table 2). Germline or somatic FAS mutations were tested in patients with selected ALPS clinical and laboratory features. One patient was lost after 1 year of follow up before the genetic analysis were performed. TACI mutations were investigated in children affected by CVID. Regarding severe hypogammaglobulinemia and B cell deficiency (with a complete lack of CD27+IgD– switched memory B cells and an expansion of the B cell subset CD21loCD38lo and CD27+IgD+ unswitched memory) of the patient with Rubinstein Taybi Syndrome, genetic analysis for mutations in BAFF-R and TACI genes were undertaken. All these gene analyses were performed by Sanger sequencing. All data were collected before the administration of immunomodulant therapies or after suspension for at least 3 months. For patients with secondary forms, data were acquired at the time of diagnosis.

**Table 1:** Patients characteristics.

	**Sex/Age at onset**	**Presenting cytopenia**	**Subsequent cytopenia**	***N* of acute episodes (average period)**	**Status at FUP**	**FUP (years)**	**Hepato spleno megaly**	**Infection**	**Others organs**	**Diagnosis (Genetics)**	**Age at dg**	**IVIG (infusions)**	**Steroids (cycles)**	**Other therapy**
1	F/12	ITP	AIHA	1	ITP	4	No		Previous inflam. diarrhea			1	Several (in other hospital)	
2	M/15	ITP + AIN	AIHA	2 (4 months)	–	1	No					2		
3	F/9	AIHA	ITP	2 (2 years)	Resolved	3	No					1	2	
4	F/8	ITP, AIHA		5 (1 years)	Resolved	7	Yes					12	2	MMF
5	F/14	ITP	AIHA	5 (4 months)	Resolved	4	No		DVT, PE, MPGN	SLE	16	1	3	
6	F/14	ITP, AIHA		4 (1 years)	ITP	4	Yes		Thyroiditis psoriasis	MCTD	15	1	4	MMF
7	M/9	ITP	AIHA	Chronic ITP	ITP	4	Yes		Thyroiditis celiac disease	CVID (TACI TNFRSF6: neg)	13	2		
8	M/2	ITP	AIHA	7 (18 months)	Resolved	14	Yes	Bronchitis and otitis (CHL), resistant HP	Allergic asthma	CVID (TACI, TNFRSF6:neg)	14	2	2	IRT
9	M/8	ITP	AIHA	4 (6 months)	Resolved	13	Yes			ALPS-FAS (TNFRSF6 mut)	9	2	3	MMF, Rituximab, IRT
10	M/2	ITP, AIHA		2 (8 years)	ITP	9	Yes	Tympanostomy tube for acute otitis media		CVID (TACI neg)	10	5	2	MMF, sirolimus, eltrombopag
11	F/5	AIN	ITP	6 (2 years)	Resolved	17	No	Bronchiectasis	Rubinsten Taybi Sdr.	Low-B RTS (BAFF-R mut)	22	2	4	IRT
12	F/13	ITP, AIHA, AIN		2 (1 month)	ITP	2	No		Vitiligo, atopic dermatitis, thyroiditis myositis	SLE	14	2		Ciclosporin per atopic dermatitis

*AIHA, Autoimmune hemolytic anemia; AIN, Autoimmune neutropenia; ALPS, Autoimmune Lymphoproliferative Syndrome; APS, Antiphospholipid Antibody Syndrome; CID, Combined Immunodeficiecy; CVID, Common Variable Immunodeficiency; CHL, Conductive Hearing Loss; DNT cell, TCRα/β+CD4–CD8– Double Negative T cell; DVT, deep vein thrombosis; HP, Helicobacter pylori; IRT, Immunoglobulin Replacement Therapy; ITP, Immune Thrombocytopenia; MCTD, Mixed Connective Tissue Disease; MMF, Mycophenolate mofetil; MPGN, Membranoproliferative Glomerulonephritis; PE, Pulmonary Embolism; SLE, Systemic Lupus Erythematosus*.

**Table 2:** Laboratory investigation.

	**Hb Nadir g/dl**	**ANC Nadir μL**	**IgG mg/dl**	**IgA mg/dl**	**IgM mg/dl**	**WBC μL**	**Lymph μL**	**CD3+ %**	**CD4+ %**	**CD4+ μL**	**CD8+ %**	**CD4+/CD8+**	**NK %**	**CD19+ %**	**CD3+ γ+δ+ %**	**CD3+DNT %**	**Fas activity**	**Auto antibodies**	**Vit B12**	**Vaccine response**
1	6.7		1,087	105	130	11,560	2,231	76	49	1,093	23	2.13	2^−^	20.9	1.7	1.6			275	
2	8.5	620	1,101	102	104	2,080^**−**^	1,248	84^**+**^	53^+^	661	23	2.30	4	10	3	2.8		ANA 1:160	855	
3	6.2		928	106	142	11,520	2,926	79^**+**^	41	1,200	34	1.21	6	14	3	1.5				
4	7.5		1,114	114	131	6,340	2,110	77	62^**+**^	1,308	32	1.94	8.5	6.5^**−**^	2	1.8			268	
5	6.9		1,596	237	171	7,380	2,347	63	36	845	22	1.64 ^+^	13	23.5^**+**^				ANA reflex 1:640, ENA/ANA 14, SS-A60+++, SS-ARo52+++, Ro60 240 U/ml, dsDNA 32 IU/ml, CLIgG 54 U/ml, CLIgM 23 U/ml	306	
6	8.5		1,807^**+**^	138	227	6,840	2,161	31^**−**^	16^**−**^	346^−^	12^−^	1.33	5	63^**+**^				ANA reflex 1:640, ENA/ANA 36, RNP70+++, RNPA++, U1RNP 201 U/ml, Tg 1967 U/ml, TPO 95 U/ml	214	
7	7.8		585^**−**^	21^**−**^	37^**−**^	5,130	1,272^**−**^	79^**+**^	52^**+**^	661	23	2.26	9	12	2	4,2	NEG	Tg 1238 UI/ml, tTG 126 U/ml	961	No
8	8.3		521^**−**^	21^**−**^	26^**−**^	5,730	2,636	87^**+**^	43	1,133	34	1.26	6	5^**−**^	2.6	3.6	NEG		994	No
9	7.2		1,590	341^**+**^	22^**−**^	3,610	1,527	76	35	535	28	1.25	13	11	7	5.6	POS		>2,000	Yes
10	6.5		440^**−**^	55^**−**^	14^**−**^	7,150	1,160^**−**^	61	31	360^−^	26	1.19	5	33.3^**+**^	6	1		ANAreflex 1:320, ENA/ANA 2.3, Sp100+++, AMA M2 +		Yes
11		329	184^**−**^	6^**−**^	8^**−**^	5,820	2,178	90^**+**^	49	1,067	36	1.36	9	0.9^**−**^	4	1.6			396	No
12	9	710	1,265	183	133	4,490^**−**^	1,440^**−**^	70	39	562	29^−^	1.34	8	20^+^	2	1		ANA reflex 1:640, ENA/ANA 6, SS-A+,Ro52++, SRP ++, dsDNA 21 IU/ml, CLIgM 557 U/ml	588	

*Nadir of Hb (Hemoglobin) and ANC (Absolute Neutrophil Count) in patients with AIHA e AIN, Anti-Sm, Anti-Smith antibodies; AMA, Anti-mitochondrial antibodies; ANA, Antinuclear antibodies; CLIgG, Cardiolipin IgG; CLIgM, Cardiolipin IgM; DGP, Deamidated gliadin peptides; DNT cell, TCRα/β+CD4–CD8– Double Negative T cell; ENA, Extractable Nuclear Antigen Antibodies; RF, Rheumatoid factor; SRP, Signal recognition particle antibodies; tTG, Transglutaminase; Tg, Thyroglobulin; TPO, Thyroid Peroxidase; ^−^: <2DS; ^+^: >2DS*.

## Results

Data of 12 patients with ES, seven females (58%) and five males (42%), referred to the Pediatric Onco-Hematology Unit of Bologna between 2002 and 2018 were retrospectively collected (Table 1). Median age at onset of cytopenias was 9 years (range 2–15 years) and median follow-up time was 4 years (range 1–17 years). At presentation multiple cytopenias were found in five children (42%). A girl was diagnosed with AIHA, ITP, and AIN, three children with AIHA and ITP and two with ITP and AIN. In 7 patients (58%), a second cytopenia developed only afterward. Five of these had primarily developed ITP and subsequently AIHA, the other two children developed ITP following AIHA or AIN. Patients experienced a median of four episodes (range 1–7), median time between acute events was 15 months (range 4 months−8 years). Six patients (50%) presented significant hepatosplenomegaly or lymphadenopathy, three children (25%) developed relevant infections (e.g., bronchiectasis pneumonia, tympanostomy tubes). Eight out of 12 (67%) patients were diagnosed for PID or a rheumatological disorder. Five children were diagnosed with PID (42%): three children with CVID (25%), one with ALPS-FAS (8%), and one child affected by Rubinstein Taybi Syndrome had developed a progressive severe B cell deficiency with hypogammaglobulinemia related to a BAFF-R mutation (low B-RTS, 8%) (22). Three were diagnosed with a rheumatological disease (two with SLE, one with MCTD). Four children (33%), one affected by CVID, also developed another autoimmune disorder (Thyroiditis, Celiac disease, Psoriasis, Vitiligo, Myositis, Membranoproliferative Glomerulonephritis).

Anti-platelet antibodies were detected in all the children and direct antiglobulin test was positive in 11/12 (82%). Only one child was found with anti-neutrophil antibodies (8%). Antinuclear antibody (ANA) positivity was detected in four patients (33%), three of which developed a rheumatological disease subsequently.

Three patients were lymphopenic (25%) all of these were diagnosed with a secondary ES. One was lost after 1 year of follow-up. None of the patients had low rate of CD4+/CD8+ T cells. In four patients (33%), a high DNT count (≥2.5% CD3+ lymphocytes) was found. One of them was diagnosed with ALPS, two with CVID, and one was lost to follow-up before performing other tests. In other three patients with high DNT count a FAS-mediated apoptosis of mitogen-stimulated T cells and gene analysis for TNFRSF6 mutation by Sanger sequencing were performed. In only one child affected by ALPS, the FAS-induced lymphocyte apoptosis assay resulted defective. In this patient germinal TNFRSF6 mutation G286X (c. 856G>T) not previously described was found.

TACI mutation was not detected in any patient with CVID. In a patient with low B-RTS, no TACI mutations were identified whereas it was detected the previously described BAFF-R monoallelic variant P21R (c.62C>G) (23).

B cell count was low in six patients (50%), four of them affected by a PIDs, and high in two girls (17%) both affected by a rheumatological disease. In six patients (50%), altered immunoglobulin levels were found. One girl affected by MCTD showed high IgG value, meanwhile four cases of PID (three CVID, one low-B RTS) revealed low immunoglobulin levels. One patient affected by ALPS showed high IgA and low IgM. The immune responses to vaccines (Tetanus toxoid) resulted normal in child affected by ALPS and absent in low-B RTS and two CVID patients. A patient with CVID maintained protective levels of antibodies in particular against the Tetanus toxoid (with absent B memory IgD+ and switched).

Cytopenias were treated as shown in Table 1. All children received first-line treatments with IVIG or corticosteroids. Three children (23%), all diagnosed with an underlying condition, underwent second-line therapies in order to control resistant ITP. One child affected by CVID was treated with Sirolimus and Eltrombopag with partial control of cytopenias. In a girl with MCTD, partial control of ITP and AHIA was achieved after Mycophenolate Mofetil therapy. Rituximab was effective in a child with ALPS even though he had developed a persistent hypogammaglobulinemia and is now undergoing immunoglobulin replacement therapy (IRT). In three children developing AIN, Granulocyte-colony stimulating factor therapy was not required. Two of these, one affected by LES, showed a trilinear cytopenia. Two episodes of ITP were treated with IGIV, whereas AIHA and AIN where mild and hadn't required therapies.

At a median follow-up time of 4 years (range 1–17), all 12 patients were alive. In only 7 (58%) good control of cytopenias was achieved. Other five (42%) revealed a chronic ITP partially responsive to second line therapy, out of these, four were diagnosed with a secondary ES. Three patients with a secondary ES (ALPS, CVID, and a patient with Rubinstein Taybi Syndrome) are undergoing immunoglobulin replacement therapy and maintained good hematological values control. Two children affected by CVID maintained protective levels of antibodies.

## Discussion

This study describes the clinical and laboratory history of 12 patients with ES treated in our Hospital in the last 17 years. Since 1980 (24), few studies have described the heterogeneity of presenting symptoms and the different underlying etiologies that came to light after an appropriate immune and rheumatological evaluation of children affected by multilineage cytopenias (2, 5, 24–26). Our data highlight ES as a possible early manifestation of an underlying immune disorder, PID or rheumatological disease, especially when cytopenias are persistent-resistant to therapy, with an early-onset or when are associated with lymphadenopathy (7, 8, 10, 23, 26–29). Particularly, for these patients, a proper laboratory investigation and a careful follow up is necessary to achieve a proper diagnosis. More than a half of our children with ES revealed an immune dysregulation (42% PID, 16% rheumatological disease). According to literature, no correlation between etiology or outcome and timing or sequencing of cytopenias presentation was found (5, 25, 26, 30). Serum vitamin B12 level was helpful in the differential diagnosis between IgG low-ALPS and DNT high-CVID (31) in two patients with hypogammaglobulinemia, lymphoproliferation, and raised DNT cell count. Our data also highlight that making a proper diagnosis is essential to ensure optimal management of therapies. For example 10 years ago, Rituximab was effective in treating cytopenias in a patient affected by ALPS but he developed a permanent hypogammaglobulinemia. In these patients other immunomodulatory therapies as mTOR inhibitors or Mycophenolate Mofetil should be preferred and splenectomy avoided for an increased risk of sepsis (32, 33). In our cohort these corticosteroid-sparing agents, used early in treatment, were efficient in two out of 12 patients, one affected by CVID and one by MCTD.

As described in literature, ES might be considered as a dysregulation of the immune system. Different mechanisms, both cellular and humoral immunity, are involved in the immune-mediated cytopenias. Since the 80s', authors tried to describe and analyze the dysregulation of the immune system reporting a usual reduction of CD4/CD8 ratio, aberrant Th1/Th2 ratio, immunoglobulin levels alterations, IL-10 and IFN-γ increase and TGF-β suppression (34, 35).

As recently resumed by Fischer et al. (6), a combination of factor can predispose patients with PID to autoimmunity as a defective negative selection of self-reactive T and B cells with high affinity, defective peripheral editing of the B-cell receptor and self-antigen–induced cell death, defective regulatory T cells or molecules, defective clearance of immune complexes and apoptotic cell bodies, gain of function of B- or T-cell activation/effector molecules, homeostatic expansion of self-reactive T lymphocytes, exacerbated production of type I interferon or IL-1. Auto-reactive immunoglobulins could be produced due to an altered B-cell regulation during maturation and tolerance induction or by an altered T–B cells interaction. Especially IgG and rarely IgA and IgM, bound to red blood cells leading to their destruction by antibody-dependent cellular cytotoxicity and/or by the activation of the complement cascade. Neutrophils are attached by antibodies that mostly binds the IgG Fc receptor type 3b (FccIIIb receptor). Also platelets could be opsonized and be prematurely removed by the reticulo-endothelial system. In ALPS, the defect of T-cell development and FAS apoptotic pathway leads to an abnormal lymphocytes survival with lymphoproliferation and secondarily affects B cells resulting in an antibody-mediated autoimmunity (4, 5, 30, 36, 37).

Despite for IVIG, an immune-modulating mechanism has been proposed (38–40) in patients with secondary ES the resistance to therapy could be related to the persistence of long-lived plasma cells secreting pathogenic autoantibodies resistant to immunomodulatory and B cell-depletion therapies (28). Our three patients with a secondary ES receiving Immunoglobulin Replacement Therapy all maintain good hematological control.

In this scenario, recent coming of Next Generation Sequencing (NGS) dramatically changed the diagnostic workup of ES. The rapid reduction in costs and analysis times of these techniques has made it possible to routinely apply them in the evaluation of the patients with ES. This helped clinicians to better characterize the genetic profile and pathological mechanisms of patients with PID with a non-specific phenotype. In particular NGS target panels represent a powerful approach and could be really efficient when the diagnostic suspicion is relatively circumscribed while Whole Exome Sequencing find an application in conditions where the phenotype overlaps more than one PID. As recently reported in literature, these techniques have allowed finding many novel mutations in patients with PID associated with ES. In particular LRBA (Lipopolysaccharide-Responsive Beige-like an Anchor protein) and CTLA-4 (Cytotoxic T Lymphocyte Antigen-4) defects were described in patients with autoimmune cytopenias, lymphoproliferation, and humoral immune deficiency (41–44). LRBA colocalize in endosomal vesicles with CTLA-4 and its deficiency increases CTLA-4 turnover. CTLA4, upregulated in activated conventional T cells and constitutively expressed in FoxP3+ regulatory ones, acts with a competitive mechanism with CD28 for binding B7 molecules (45). In patients with increased frequency of infections, autoimmune cytopenias and lymphoid hyperplasia these techniques allowed discovery a gain-of-function of PI-3-kinase, activated downstream of CD28 ligation and involved in B-cell proliferation and survival signaling (45, 46), or that of STAT3, involved in the suppression of Treg function and cytokine signaling (45, 47). Furthermore, thanks to Whole Exome Sequencing a homozygous mutation of TPP2, a postproteaosomal cytosolic protease, were found in 6 patients with early-onset Evans syndrome, respiratory infection, and developmental delay (48, 49). For some of these genetic mutations, targeted therapies with a reduced toxicity profile are under investigation for the opportunity of improving outcome (11).

Due to the rare nature of pediatric ES and refractory cytopenias, collaborative studies are necessary to investigate how each genetic mutation and specific pathogenetic mechanism is correlated to different clinical pictures. Thus, patients need to be included in national observational studies (25, 29, 30) or ideally the cohort should be extended to an international perspective (www.sic-reg.org). This management could help us to determine a structured diagnostic algorithm, genetic tools and therapeutic strategies.

**Figure 1:**
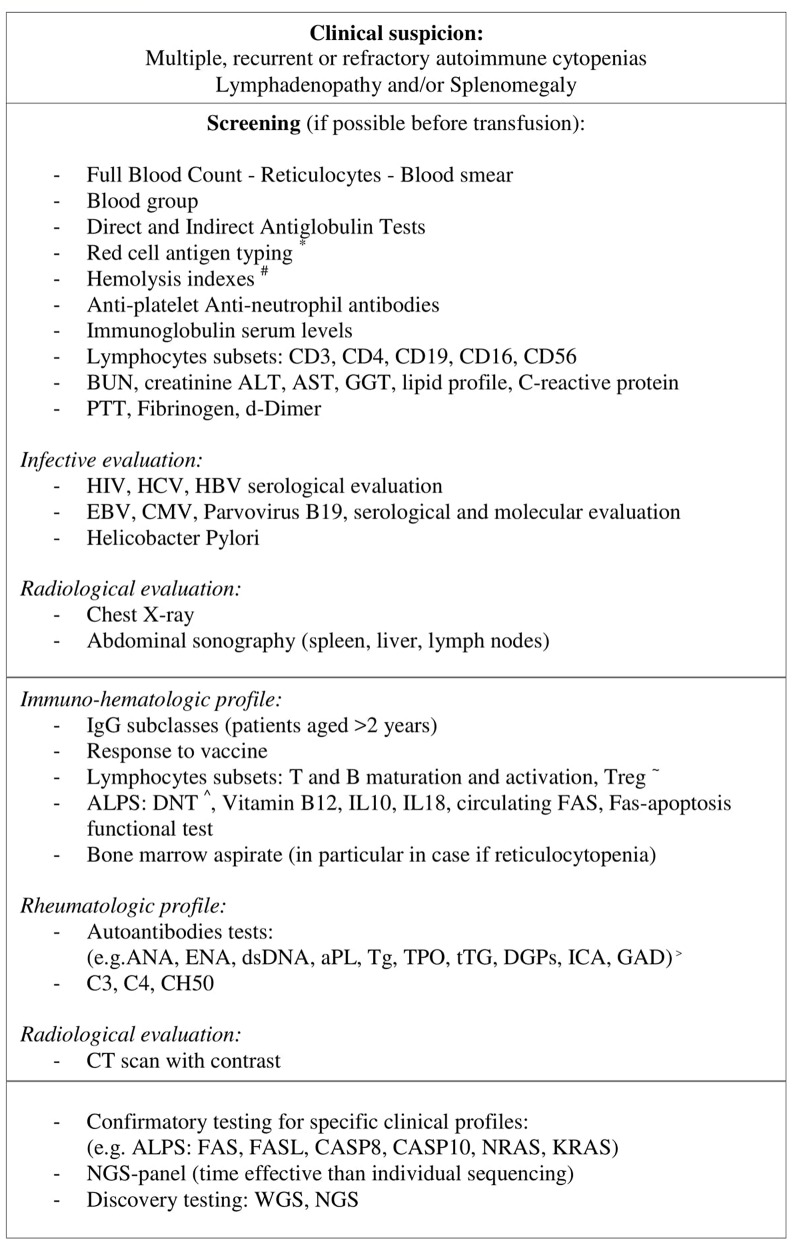
In patients with multiple, recurrent, or refractory autoimmune cytopenias and a high clinical suspicion for an underlying disorder if general screening gives positive result further laboratory and radiological analysis should be performed. When a particular clinical suspicious is identified a specific genetic test can be done. In patients with a non-specific profile, considering the declining cost and execution time of new gene sequencing techniques (NGS panel, WES, and NGS), these methodologies are likely to be more time and cost-effective than individual sequencing. *Red cell antigen typing: C, c, D, E, e, K, Jka, Jkb, Fya, Fyb, S, s, ^#^Hemolysis indexes: LDH, haptoglobin, bilirubin, ^~^ T and B maturation and activation: CD4/CD8 T naive (CD45RA+), CD4/CD8 T memory (CD45RO), B naive (CD27–), B memory (CD27+), BCD21loCD38lo, Treg(CD4+CD25+CD127(low/–)), ^∧^DNT: CD3+CD4-CD8-alfa/beta+, ^>^ ANA, anti-nuclear; ENA, anti-extractable nuclear antigen; aPL, anti-phospholipid; Tg, Thyroglobulin; TPO, Thyroid peroxidase; tTg, tissue transglutaminase IgA and IgG; DGPs, deamidated gliadin peptides; ICA, Autoantibodies against islet cells; GAD, Glutamic acid decarboxylase.

In conclusion in more than a half of our children, ES has been earliest manifestation of an underlying immune disorder, in particular when cytopenias were persistent-resistant to therapy, with early onset or associated with lymphadenopathy. We highlighted the importance of a proper laboratory-radiological investigation (Figure 1) and careful follow up of these patients in order to achieve a proper diagnosis and found the best therapeutic strategies. Further investigations are needed to understand the underlying immune dysregulation mechanisms, define markers of disease severity and the benefit–risk profile of second-line therapies.

## Ethics Statement

Written informed consent for the publication of this retrospective paper were obtained from the parents.

## Author Contributions

BR and GP collected the data. BR and DZ analyzed the data and participated in writing the paper. EF, MC, AM, APr, and APe reviewed the article. All authors were involved in the clinical care of the patients, critically read the manuscript, approved the final version, and agreed to be accountable for all aspects of the work.

### Conflict of Interest Statement

The authors declare that the research was conducted in the absence of any commercial or financial relationships that could be construed as a potential conflict of interest.

## Footnotes

^1^https://esid.org/Media/Files/Common-Variable-Immunodeficiency-diagnostic-criteria

## Abbreviations

AIHA: Autoimmune Hemolytic Anemia
AIN: Autoimmune Neutropenia
ALPS: Autoimmune Lymphoproliferative Syndrome
ANA: Antinuclear Antibody
APS: Antiphospholipid Antibody Syndrome
CID: Combined Immunodeficiency
CVID: Common Variable Immunodeficiency
DNT cell: TCRα/β+CD4–CD8– Double Negative T cell
ES: Evans Syndrome
Hb: Hemoglobin
IRT: Immunoglobulin Replacement Therapy
ITP: Immune Thrombocytopenia
Low-B RTS: Rubinsten Taybi Syndrome With a Progressive Severe B Cell Deficiency and Hypogammaglobulinemia
MCTD: Mixed Connective Tissue Disease
NGS: Next Generation Sequencing
PID: Primary Immunodeficiencies
SLE: Systemic Lupus Erythematosus
WES: Whole Exome Sequencing.

## References

[1] Evans RS Takahashi K Duane RT Payne R Liu C. Primary thrombocytopenic purpura and acquired hemolytic anemia; evidence for a common etiology. AMA Arch Intern Med. (1951) 87:48–65. 10.1001/archinte.1951.03810010058005 14782741

[2] Mathew P Chen G Wang W. Evans syndrome: results of a national survey. J Pediatr Hematol Oncol. (1997) 19:433–7. 10.1097/00043426-199709000-00005 9329465

[3] Wang WC. Evans syndrome in childhood: pathophysiology, clinical course, and treatment. J Pediatr Hematol Oncol. (1988) 10:330–8. 10.1097/00043426-198824000-00013 3071168

[4] Miano M. How I manage Evans syndrome and AIHA cases in children. Br J Haematol. (2016) 172:524–34. 10.1111/bjh.13866 26625877

[5] Mantadakis E Farmaki E. Natural history, pathogenesis, and treatment of Evans syndrome in children. J Pediatr Hematol Oncol. (2017) 39:413–9. 10.1097/MPH.0000000000000897 28654461

[6] Fischer A Provot J Jais J-P Alcais A Mahlaoui N Adoue D. Autoimmune and inflammatory manifestations occur frequently in patients with primary immunodeficiencies. J Allergy Clin Immunol. (2017) 140:1388–1393.e8. 10.1016/j.jaci.2016.12.978 28192146

[7] Notarangelo LD Primary immunodeficiencies (PIDs) presenting with cytopenias. Hematol Am Soc Hematol Educ Progr. (2009) 1:139–43. 10.1182/asheducation-2009.1.139 20008192

[8] Martínez-Valdez L Deyà-Martínez A Giner MT Berrueco R Esteve-Solé A Juan M. Evans syndrome as first manifestation of primary immunodeficiency in clinical practice. J Pediatr Hematol Oncol. (2017) 39:490–4. 10.1097/MPH.0000000000000880 28937520

[9] Michel M Chanet V Dechartres A Morin A-S Piette J-C Cirasino L. The spectrum of Evans syndrome in adults: new insight into the disease based on the analysis of 68 cases. Blood. (2009) 114:3167–72. 10.1182/blood-2009-04-215368 19638626

[10] Teachey DT Lambert MP. Diagnosis and management of autoimmune cytopenias in childhood. Pediatr Clin North Am. (2013) 60:1489–511. 10.1016/j.pcl.2013.08.009 24237984 PMC5384653

[11] Rotz SJ Ware RE Kumar A. Diagnosis and management of chronic and refractory immune cytopenias in children, adolescents, and young adults. Pediatr Blood Cancer. (2018) 65:e27260. 10.1002/pbc.27260 29856527

[12] Rodeghiero F Stasi R Gernsheimer T Michel M Provan D Arnold DM. Standardization of terminology, definitions and outcome criteria in immune thrombocytopenic purpura of adults and children: report from an international working group. Blood. (2009) 113:2386–93. 10.1182/blood-2008-07-162503 19005182

[13] Bass GF Tuscano ET Tuscano JM. Diagnosis and classification of autoimmune hemolytic anemia. Autoimmun Rev. (2014) 13:560–4. 10.1016/j.autrev.2013.11.010 24418298

[14] Youinou P Jamin C Le Pottier L Renaudineau Y Hillion S Pers J-O. Diagnostic criteria for autoimmune neutropenia. Autoimmun Rev. (2014) 13:574–6. 10.1016/j.autrev.2014.01.001 24418296

[15] Oliveira JB Bleesing JJ Dianzani U Fleisher TA Jaffe ES Lenardo MJ. Revised diagnostic criteria and classification for the autoimmune lymphoproliferative syndrome (ALPS): report from the 2009 NIH International Workshop. Blood. (2010) 116:e35–e40. 10.1182/blood-2010-04-280347 20538792 PMC2953894

[16] Cappelli S Bellando Randone S Martinović D Tamas M-M Pasalić K Allanore Y To be or not to be, 10 years after: evidence for mixed connective tissue disease as a distinct entity. Semin Arthritis Rheum. (2012) 41:589–98. 10.1016/j.semarthrit.2011.07.010 21959290

[17] Petri M Orbai A-M Alarcón GS Gordon C Merrill JT Fortin PR. Derivation and validation of the Systemic Lupus International Collaborating Clinics classification criteria for systemic lupus erythematosus. Arthritis Rheum. (2012) 64:2677–86. 10.1002/art.34473 22553077 PMC3409311

[18] Lythgoe H Morgan T Heaf E Lloyd O Al-Abadi E Armon K. Evaluation of the ACR and SLICC classification criteria in juvenile-onset systemic lupus erythematosus: a longitudinal analysis. Lupus. (2017) 26:1285–90. 10.1177/0961203317700484 28361566

[19] Teachey DT. Unmasking Evans syndrome: T-cell phenotype and apoptotic response reveal autoimmune lymphoproliferative syndrome (ALPS). Blood. (2005) 105:2443–8. 10.1182/blood-2004-09-3542 15542578

[20] Seif AE Manno CS Sheen C Grupp SA Teachey DT. Identifying autoimmune lymphoproliferative syndrome in children with Evans syndrome: a multi-institutional study. Blood. (2010) 115:2142–5. 10.1182/blood-2009-08-239525 20068224

[21] Bleesing JJH. Immunophenotypic profiles in families with autoimmune lymphoproliferative syndrome. Blood. (2001) 98:2466–73. 10.1182/blood.V98.8.2466 11588044

[22] Lougaris V Facchini E Baronio M Lorenzini T Moratto D Specchia F. Progressive severe B cell deficiency in pediatric Rubinstein-Taybi syndrome. Clin Immunol. (2016) 173:181–3. 10.1016/j.clim.2016.10.019 27825976

[23] Pieper K Rizzi M Speletas M Smulski CR Sic H Kraus H. A common single nucleotide polymorphism impairs B-cell activating factor receptor's multimerization, contributing to common variable immunodeficiency. J Allergy Clin Immunol. (2014) 133:1222–5. 10.1016/j.jaci.2013.11.021 24406071

[24] Pui C-H Wilimas J Wang W. Evans syndrome in childhood. J Pediatr. (1980) 97:754–8. 10.1016/S0022-3476(80)80258-7 7191890

[25] Aladjidi N Fernandes H Leblanc T Vareliette A Rieux-Laucat F Bertrand Y. Evans syndrome in children: long-term outcome in a prospective French National Observational Cohort. Front Pediatr. (2015) 3:79. 10.3389/fped.2015.00079 26484337 PMC4586429

[26] Al Ghaithi I Wright NAM Breakey VR Cox K Warias A Wong T. Combined autoimmune cytopenias presenting in childhood. Pediatr Blood Cancer. (2016) 63:292–8. 10.1002/pbc.25769 26397379

[27] Antoon JW Metropulos D Joyner BL. Evans syndrome secondary to common variable immune deficiency. J Pediatr Hematol Oncol. (2016) 38:243–5. 10.1097/MPH.0000000000000550 26950085

[28] Ghosh S Seidel MG. Editorial: current challenges in immune and other acquired cytopenias of childhood. Front Pediatr. (2016) 4:3. 10.3389/fped.2016.00003 26870718 PMC4735444

[29] Sipurzynski J Fahrner B Kerbl R Crazzolara R Jones N Ebetsberger G. Management of chronic immune thrombocytopenia in children and adolescents: lessons from an Austrian national cross-sectional study of 81 patients. Semin Hematol. (2016) 53:S43–S47. 10.1053/j.seminhematol.2016.04.013 27312164

[30] Aladjidi N Leverger G Leblanc T Picat MQ Michel G Bertrand Y. New insights into childhood autoimmune hemolytic anemia: a French national observational study of 265 children. Haematologica. (2011) 96:655–63. 10.3324/haematol.2010.036053 21228033 PMC3084911

[31] Rensing-Ehl A Warnatz K Fuchs S Schlesier M Salzer U Draeger R. Clinical and immunological overlap between autoimmune lymphoproliferative syndrome and common variable immunodeficiency. Clin Immunol. (2010) 137:357–65. 10.1016/j.clim.2010.08.008 20832369

[32] Rao VK Price S Perkins K Aldridge P Tretler J Davis J. Use of rituximab for refractory cytopenias associated with autoimmune lymphoproliferative syndrome (ALPS). Pediatr Blood Cancer. (2009) 52:847–52. 10.1002/pbc.21965 19214977 PMC2774763

[33] Rao VK Oliveira JB. How I treat autoimmune lymphoproliferative syndrome. Blood. (2011) 118:5741–51. 10.1182/blood-2011-07-325217 21885601 PMC3228494

[34] Wang W Herrod H Pui C-H Presbury G Wilimas J. Immunoregulatory abnormalities in evans syndrome. Am J Hematol. (1983) 15:381–90. 10.1002/ajh.2830150409 6606357

[35] Karakantza M Mouzaki A Theodoropoulou M Bussel JB Maniatis A. Th1 and Th2 cytokines in a patient with Evans' syndrome and profound lymphopenia. Br J Haematol. (2000) 110:968–70. 10.1046/j.1365-2141.2000.02296.x 11054090

[36] Farruggia P Dufour C. Diagnosis and management of primary autoimmune neutropenia in children: insights for clinicians. Ther Adv Hematol. (2015) 6:15–24. 10.1177/2040620714556642 25642312 PMC4298488

[37] Seidel MG. Autoimmune and other cytopenias in primary immunodeficiencies: pathomechanisms, novel differential diagnoses, and treatment. Blood. (2014) 124:2337–44. 10.1182/blood-2014-06-583260 25163701 PMC4192747

[38] Anthony RM Kobayashi T Wermeling F Ravetch JV. Intravenous gammaglobulin suppresses inflammation through a novel T H 2 pathway. Nature. (2011) 475:110–4. 10.1038/nature10134 21685887 PMC3694429

[39] Nagelkerke SQ Dekkers G Kustiawan I van de Bovenkamp FS Geissler J Plomp R. Inhibition of FcγR-mediated phagocytosis by IVIg is independent of IgG-Fc sialylation and FcγRIIb in human macrophages. Blood. (2014) 124:3709–18. 10.1182/blood-2014-05-576835 25352126

[40] Burns JC Franco A. The immunomodulatory effects of intravenous immunoglobulin therapy in Kawasaki disease. Expert Rev Clin Immunol. (2015) 11:819–25. 10.1586/1744666X.2015.1044980 26099344 PMC4985263

[41] Kuehn HS Ouyang W Lo B Deenick EK Niemela JE Avery DT. Immune dysregulation in human subjects with heterozygous germline mutations in CTLA4. Science. (2014) 345:1623–7. 10.1126/science.1255904 25213377 PMC4371526

[42] Schubert D Bode C Kenefeck R Hou TZ Wing JB Kennedy A. Autosomal dominant immune dysregulation syndrome in humans with CTLA4 mutations. Nat Med. (2014) 20:1410–6. 10.1038/nm.3746 25329329 PMC4668597

[43] Revel-Vilk S Fischer U Keller B Nabhani S Gámez-Díaz L Rensing-Ehl A. Autoimmune lymphoproliferative syndrome-like disease in patients with LRBA mutation. Clin Immunol. (2015) 159:84–92. 10.1016/j.clim.2015.04.007 25931386

[44] Besnard C Levy E Aladjidi N Stolzenberg M-C Magerus-Chatinet A Alibeu O. Pediatric-onset Evans syndrome: heterogeneous presentation and high frequency of monogenic disorders including LRBA and CTLA4 mutations. Clin Immunol. (2018) 188:52–7. 10.1016/j.clim.2017.12.009 29330115

[45] Grimbacher B Warnatz K Yong PFK Korganow A. The crossroads of autoimmunity and immunodeficiency : lessons from polygenic traits and monogenic defects. J Allergy Clin Immunol. (2017) 137:3–17. 10.1016/j.jaci.2015.11.004 26768758

[46] Lucas CL Kuehn HS Zhao F Niemela JE Deenick EK Palendira U. Dominant-activating germline mutations in the gene encoding the PI(3)K catalytic subunit p110δ result in T cell senescence and human immunodeficiency. Nat Immunol. (2014) 15:88–97. 10.1038/ni.2771 24165795 PMC4209962

[47] Milner JD Vogel TP Forbes L Ma CA Stray-Pedersen A Niemela JE. Early-onset lymphoproliferation and autoimmunity caused by germline STAT3 gain-of-function mutations. Blood. (2015) 125:591–9. 10.1182/blood-2014-09-602763 25359994 PMC4304103

[48] Lu W Zhang Y McDonald DO Jing H Carroll B Robertson N. Dual proteolytic pathways govern glycolysis and immune competence. Cell. (2014) 159:1578–90. 10.1016/j.cell.2014.12.001 25525876 PMC4297473

[49] Stepensky P Rensing-Ehl A Gather R Revel-Vilk S Fischer U Nabhani S. Early-onset Evans syndrome, immunodeficiency, and premature immunosenescence associated with tripeptidyl-peptidase II deficiency. Blood. (2015) 125:753–61. 10.1182/blood-2014-08-593202 25414442 PMC4463807

